# A molecular study of pediatric pilomyxoid and pilocytic astrocytomas: Genome-wide copy number screening, retrospective analysis of clinicopathological features and long-term clinical outcome

**DOI:** 10.3389/fonc.2023.1034292

**Published:** 2023-02-13

**Authors:** Essam AlShail, Ahmed Nasser Alahmari, Anas A. M. Dababo, Maysoon Alsagob, Hindi Al-Hindi, Hala Khalil, Zainab Al Masseri, Razan AlSalamah, Ethar Almohseny, Amjad Alduhaish, Dilek Colak, Namik Kaya

**Affiliations:** ^1^ Department of Neurosciences, King Faisal Specialist Hospital and Research Centre (KFSHRC), Riyadh, Saudi Arabia; ^2^ Department of Pathology and Laboratory Medicine, King Faisal Specialist Hospital and Research Centre (KFSHRC), Riyadh, Saudi Arabia; ^3^ Department of Translational Genomics, Center for Genomic Medicine, King Faisal Specialist Hospital and Research Centre (KFSHRC), Riyadh, Saudi Arabia; ^4^ Applied Genomics Technologies Institute, King Abdulaziz City for Science and Technology, Riyadh, Saudi Arabia; ^5^ Department of Biostatistics, Epidemiology and Scientific Computing, King Faisal Specialist Hospital and Research Centre (KFSHRC), Riyadh, Saudi Arabia; ^6^ Medical Genetics Department, King Faisal Specialist Hospital and Research Centre (KFSHRC), Riyadh, Saudi Arabia; ^7^ Neuroscience Department, King Abdullah Medical City, Mecca, Saudi Arabia; ^8^ Department of Molecular Oncology, King Faisal Specialist Hospital and Research Centre (KFSHRC), Riyadh, Saudi Arabia

**Keywords:** pilomyxoid astrocytoma, *BRAF*, *KIAA1549*, copy number alteration, pilocytic astrocytoma, genomics, brain tumor, gene network

## Abstract

**Background:**

Pilocytic Astrocytoma (PA) is the most common pediatric brain tumors. PAs are slow-growing tumors with high survival rates. However, a distinct subgroup of tumors defined as pilomyxoid astrocytoma (PMA) presents unique histological characteristics and have more aggressive clinical course. The studies on genetics of PMA are scarce.

**Methods:**

In this study, we report one of the largest cohort of pediatric patients with pilomyxoid (PMA) and pilocytic astrocytomas (PA) in Saudi population providing a comprehensive clinical picture, retrospective analysis with long-term follow-up, genome-wide copy number changes, and clinical outcome of these pediatric tumors. We examined and compared genome-wide copy number aberrations (CNAs) and the clinical outcome of the patients with PA and PMA.

**Results:**

The median progression free survival for the whole cohort was 156 months and it was 111 months for the PMA, however, not statistically significantly different between the groups (log-rank test, P = 0.726). We have identified 41 CNAs (34 gains and 7 losses) in all tested patients. Our study yielded the previously reported KIAA1549-BRAF Fusion gene in over 88% of the tested patients (89% and 80% in PMA and PA, respectively). Besides the fusion gene, twelve patients had additional genomic CNAs. Furthermore, pathway and gene network analyses of genes in the fusion region revealed alterations in retinoic acid mediated apoptosis and MAPK signaling pathways and key hub genes that may potentially be involved in tumor growth and progression, including *BRAF*, *LUC7L2*, *MKRN1*, *RICTOR*, *TP53*, *HIPK2*, *HNF4A*, *POU5F*, and *SOX4*.

**Conclusion:**

Our study is the first report of a large cohort of patients with PMA and PA in the Saudi population that provides detailed clinical features, genomic copy number changes, and outcome of these pediatric tumors and may help better diagnosis and characterization of PMA.

## Introduction

Brain and other central nervous system related tumors are the largest group of childhood cancers and the leading cause of cancer-related death in children. Among these, pilocytic astrocytomas (PAs) are the most frequently encountered tumors with peak incidence in the first decade of life (1, 2). Auspiciously, PAs are not aggressive, typically slow growing brain tumors and have a much better prognosis compared to other more aggressive neoplasms. These tumors are considered being grade I tumors according to the WHO (World Health Organization), and progression to higher grades is quite unusual. Patients with PA have high survival rates and only a small percentage of the patients demonstrate a variable clinical course with decreased length of disease-free survival and higher mortality rates (3). However, a distinct subtype of tumors termed pilomyxoid astrocytomas (PMAs) displays more aggressive clinical course and higher recurrence rate (4). The PMAs prefer the optic-chiasmatic/hypothalamic region, however, there are several studies reporting PMAs in several other locations (5). In addition, some tumors present transitional morphological features with combined pilocytic and pilomyxoid features (mixed) (3). The PMAs were previously considered as WHO grade II tumor (4, 5).

Previous reports have shown that about two thirds of PAs display a recurrent gain at 7q34 (6, 7). This leads to gain of *BRAF*, with subsequent BRAF activation. Another study indicated that low-grade gliomas have distinct copy number gains at chr6q23 (8). Interestingly, genetic changes may vary among PA, PMA, and IPT (intermediate pilomyxoid tumors). For instance, among 15 patients only 3 had *KIAA1549-BRAF*-fusion and 2 had *BRAF* ^*V*600*E*^-mutation whereas six patients had wild type *BRAF* (9). Similarly, it was reported that there was significant difference in the gene expression patterns between PA vs PMA (10). Compared to the general knowledge on PAs, very little is known about the molecular characteristics of PMAs. The molecular mechanisms that may explain PMA’s more aggressive behavior are still lacking. In this study, we examined and compared the clinical features and outcome in a large cohort of the PA and PMA patients, majority of which were PMAs. We also delineated genetic characteristics using genomic arrays and identified copy number changes associated with PMA and PA.

## Materials and methods

### Tumor samples

We performed a retrospective chart review study of selected patients (n=27) who were histologically determined to have pilomyxoid (PMA) and pilocytic astrocytomas (PA) who were diagnosed between November 1998 and March 2015 at King Faisal Specialist Hospital and Research Centre (KFSHRC). The patients’ charts were reviewed until March 07, 2021. The study was performed under a locally approved IRB (King Faisal Specialist Hospital and Research Centre, Research Advisory Council, Basic Research as well as Ethics Committees, RAC# 2111055) and fulfilled the principles of the Helsinki Declaration, 2013. The histopathological assessment and determination of the tumors were done by neuropathologists at KFSHRC based on microscopic, morphological, and immunohistochemical characteristics of the tumors (5, 11, 12). Among the 27 patients, 17 of them were cytogenetically examined using OncoScan arrays.

### DNA isolation

Samples were obtained from the FFPE blocks as several microtome shaves; each is around 5 to 6 μm thick. DNA was extracted using QIAamp DNA FFPE Tissue Kit (Qiagen, Hilden, Germany) or similar kits based on the Affymetrix’s recommended protocols. DNA was quantified *via* two different systems Quant-iT (Thermo Fisher, Waltham, Massachusetts, US) and Bioanalyzer (Agilent Technologies, Santa Clara, US). The quality and quantity of the DNA samples were evaluated by assessing the results from both systems.

### Genechip OncoScan arrays

OncoScan arrays and related assays were used for detection of chromosomal abnormalities. The array workflow is based on company’s protocols (Affymetrix, Santa Clara, CA, US). The FFPE DNA sample preparation, array hybridization, as well as subsequent reactions such as amplification and labeling were strictly followed according to the assay user guide (P/N 703175). The labeled samples were hybridized to the arrays, washed and scanned using Affymetrix scanner. The data was analyzed using Affymetrix’s Chromosome Analysis Suite (ChAS) using the default settings (high resolution) of the chromosomal abnormality detection.

### PCR

We utilized some of the remaining DNA from FFPE samples during DNA amplification. PCR was carried out using the Qiagen HotStar Tag (Life Technologies, USA) according to standard protocols. The products were visually inspected by agarose gel electrophoresis. Afterwards, PCR amplicons were sequenced using the BigDye Terminator reaction mix and run on the Sequencing equipment (Applied Biosystems’ 3730 Sequence Analyzer, Thermo Fisher).

### Sanger sequencing

To determine the absence or presence of several known mutations Sanger sequencing (SS) was utilized. Particular mutations, including hotspot mutations in *TP53*, *PIK3CA*, *KRAS*, *EGFR*, *PTEN*, *BRAF*, *IDH1*, and *NRAS*, were picked up (**Supplementary Table 1**) as these are already targeted by OncoScan arrays using molecular inversion probes (MIP). The Sanger sequencing (SS) was carried out as a confirmatory approach to interrogate these mutations. Gene/mutation specific primers (GSP) were used in the PCRs. These primers were modified for SS (M13 universal primer sites were added to the GSP and each primer was used in separate reactions for SS).

### Functional pathway and network analysis

We performed gene network and functional analysis using Ingenuity Pathways Analysis (IPA) (QIAGEN Inc., https://www.qiagenbioinformatics.com/products/ingenuity-pathway-analysis). We also performed gene ontology (GO) enrichment analysis using Database for Annotation, Visualization and Integrated Discovery (DAVID) (13) and Protein ANalysis Through Evolutionary Relationships (PANTHER™) classification systems (14). The genes affected by copy number changes were mapped in the Ingenuity pathway knowledge base. A right-tailed Fisher’s exact test was used to calculate a p-value determining the probability that the biological function (or pathway) assigned to that data set is explained by chance alone.

### Statistical analysis

Comparison of the patients’ characteristics by tumor type (PMA, PA, and others) was performed using non-parametric Kruskal- Wallis test for continuous variables and Fisher’s exact test for categorical variables. Kaplan–Meier survival curves for each tumor type were generated to estimate the progression-free time over the observation period, followed by log-rank test to assess differences between the groups. Statistical analysis was performed using IBM SPSS Statistics Version 26.0. All statistical tests were two-sided and p-value < 0.05 was considered statistically significant.

## Results

### Clinical features of pilomyxoid and pilocytic astrocytoma patients

**Figure 1:**
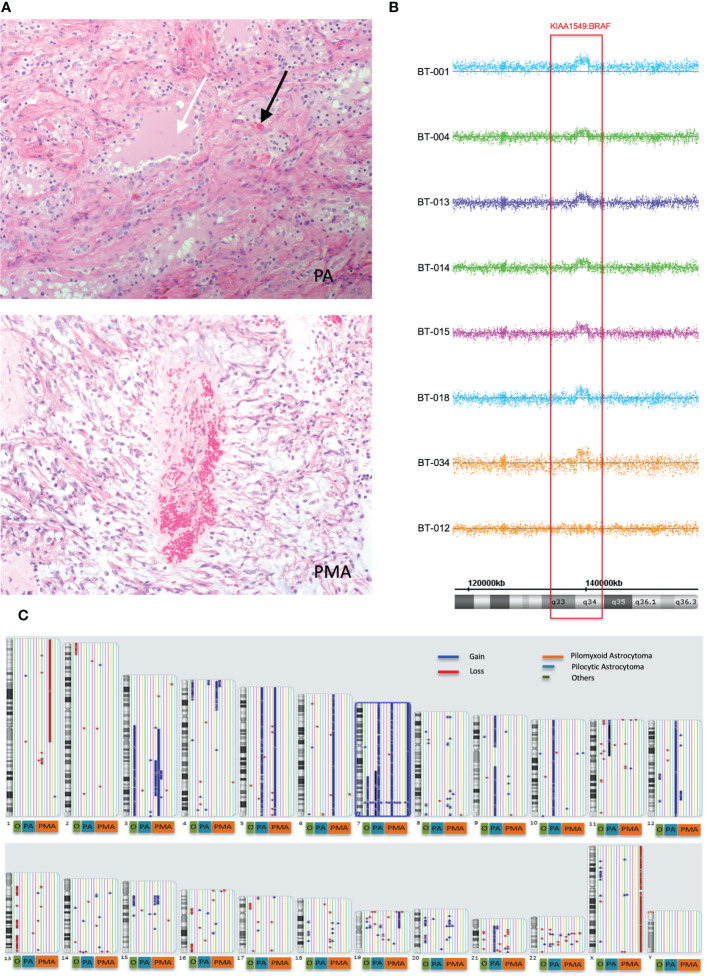
Global copy number analyses of pediatric pilomyxoid and pilocystic astrocytomas. **(A)** Representative histopathological images of pilocytic astrocytoma (PA) and pilomyxoid astrocytoma (PMA) (original magnification, 200×). The PA shows biphasic pattern with microcystic changes (white arrow) and Rosenthal fibers (black arrows) and the PMA shows perivascular arrangement with myxoid background (bluish color) and the mononmorphic piloid cells without Rosenthal fibers **(B)** Global copy number analyses of PMA samples, displaying the KIAA1549:BRAF fusion gene. Each sample is color coded. The baseline is represented as a straight line while the region consisting the fusion gene is pointed out by raised probes above the baseline (indicating the gain). The red block covers the region containing the fusion gene/gain. Below the figure, schematic chromosome bands and coordinates on chromosome 7 are illustrated. **(C)** Global copy number analyses results for all samples tested (PMA, PA and others). Each chromosome and related cytobands are represented in separate boxes. Next to each chromosome displays all samples with different colors. The samples are ordered as PMA, PA, and “others” from right to left next to the chromosome number; with orange, blue and red boxes, respectively, underneath the respective samples. The gains and losses are indicated as blue and red lines/arrow heads, respectively.

We first retrospectively reviewed the clinical and tumor characteristics of 27 pediatric patients who were diagnosed with PMA, PA, angiocentric glioma (AG), and others (intermediate tumor with anaplastic features (IAT). Some representative histopathological images of pilocytic astrocytoma (PA) and pilomyxoid astrocytoma (PMA) are shown in **Figure 1A**. The median follow-ups for PMA and PA were 60 months and 99 months, respectively. The most common location for PMA was suprasellar area (67%). The clinical and tumor characteristics of the patient cohort that we studied are summarized in **Table 1**.

**Table 1:** Characteristics of patients and tumors by tumor type.

	Total (n=27)	PMA (n=15)	PA (n=7)	Others* (n=5)	p-value
Age at diagnosis (years), median (min-max)	**2 (0.9-9)**	2 (0.9-9)	4 (0.9-7)	2 (0.9-4)	0.684
Follow-up duration (months), median (min-max)	**88 (11-367)**	60 (11-132)	98.5 (24-240)	102 (76-367)	0.069
Gender, n (%)					
*Male*	13 (48.0)	8 (53.3)	4 (57.1)	1 (20.0)	0.508
*Female*	14 (52.0)	7 (46.7)	3 (42.9)	4 (80.0)	
Tumor characteristics					
Tumor location, n (%)					
*Suprasellar region*	14 (51.9)	10 (66.7)	1 (14.3)	3 (60.0)	**0.040**
*Brainstem*	1 (3.7)	0	0	1 (20.0)	
*Cerebellum*	1 (3.7)	1 (6.7)	0	0	
*Chiasm/hypothalmus*	3 (11.1)	1 (6.7)	2 (28.6)	0	
*Left temporal*	1 (3.7)	1 (6.7)	0	0	
*Left thalamus*	1 (3.7)	0	0	1 (20.0)	
*Optic nerve*	2 (7.4)	0	2 (28.6)	0	
*Pineal region*	1 (3.7)	0	1 (14.3)	0	
*Sella/suprasellar*	1 (3.7)	0	1 (14.3)	0	
*Spinal cord*	1 (3.7)	1 (6.7)	0	0	
*Suprasellar/hypothalamic*	1 (3.7)	1 (6.7)	0	0	
Lesion type, n (%)					
*Solid*	9 (36.0)	5 (38.5)	3 (42.9)	1 (20.0)	0.926
*Mixed*	10 (40.0)	5 (38.5)	2 (28.6)	3 (60.0)	
*Cystic*	6 (24.0)	3 (23.1)	2 (28.6)	1 (20.0)	
Pattern of enhancement, n (%)					
*Homogeneous*	5 (20.0)	1 (7.7)	4 (57.1)	0	**0.016**
*Heterogeneous*	19 (76.0)	12 (92.3)	3 (42.9)	4 (80.0)	
*Non-enhancing*	1 (4.0)	0	0	1 (20.0)	
Metastasis, n (%)					
*No*	23 (85.5)	11 (78.6)	7 (100)	5 (100)	0.392
*Yes*	3 (11.5)	3 (21.4)	0	0	

*n=3 IAT, n=1 atypical glioma, n=1 AG. AG, Angiocentric Glioma; IAT, Intermediate with Anaplastic Transformation; PA, Pilocytic Astrocytoma; PMA, Pilomyxoid Astrocytoma. Bold indicates statistical significance.

All patients underwent surgeries, with earliest PMA surgery performed in 1998 and all other surgeries were performed from 1998 through 2015, that included unilateral subfrontal, bilateral subfrontal, transcortical transventricular, pterional, temporal or combined subfrontal and pterional approaches. Three-quarters of our patients underwent debulking or subtotal resection (**Table 2**). Twelve PMA patients (86%) received chemotherapy. Twenty-one patients (80%) received chemotherapy with low-grade glioma protocol consisting of vincristine and carboplatin. The Temozolamide was used in recurrent or resistant cases. Radiation treatment was used for 10 patients (38%). There was only one mortality in a patient with atypical glioma who died after two weeks due to rapid progression of the disease. Three patients had metastases (one to CSF), and all were PMAs.

**Table 2:** Management and outcomes after treatment by tumor type.

		Total (n=27)		PMA (n=15)		PA (n=7)	Others* (n=5)	p-value
Management		n (%)		n (%)		n (%)	n (%)	
Surgery approach								
*Bifrontal craniotomy*		7 (26.9)		3 (21.4)		3 (42.9)	1 (20.0)	0.988
*Cervical laminoplasty and biopsy*		1 (3.8)		1 (7.1)		0	0	
*Frontal craniotomy*		8 (30.8)		4 (28.6)		2 (28.6)	0	
*Pterional craniotomy*		5 (19.2)		3 (21.4)		1 (14.3)	3 (60.0)	
*Subfrontal craniotomy*		2 (7.7)		2 (14.3)		0	0	
*Suboccipital craniotomy*		3 (11.5)		1 (7.1)		1 (14.3)	1 (20.0)	
Extent of resection								
*Biopsy*		2 (8.0)		2 (15.4)		0	0	0.262
*D*		11 (44.0)		7 (53.8)		3 (42.9)	1 (20.0)	
*NTR*		4 (16.0)		2 (15.4)		2 (28.6)	0	
*SR*		8 (32.0)		2 (15.4)		2 (28.6)	4 (80.0)	
Ventriculoperitoneal shunt (VPS)								
*No*		8 (32.0)		3 (21.4)		3 (50.0)	2 (40.0)	0.435
*Yes*		17 (68.0)		11 (78.6)		3 (50.0)	3 (60.0)	
Radiotherapy								
*No*		16 (61.5)		9 (64.3)		4 (57.1)	3 (60.0)	1.00
*Yes*		10 (38.5)		5 (35.7)		3 (42.9)	2 (40.0)	
Chemotherapy								
*No*		5 (19.2)		2 (14.3)		3 (42.9)	0	0.210
*Yes*		21 (80.8)		12 (85.7)		4 (57.1)	5 (100)	
Redo surgery								
*No*		22 (84.6)		12 (85.7)		6 (85.7)	4 (80.0)	1.00
*Yes*		4 (15.4)		2 (14.3)		1 (14.3)	1 (20.0)	
Outcomes after treatment								
Visual impairment								
*Blindness*		4 (16.7)		3 (21.4)		0	1 (20.0)	0.266
*Decrease visual acuity*		3 (12.5)		0		2 (28.6)	1 (20.0)	
*Eye squint*		1 (4.2)		0		0	1 (20.0)	
*Left eye blindness*		1 (4.2)		0		1 (14.3)	0	
*Right eye blindness*		3 (12.5)		2 (14.3)		1 (14.3)	0	
*NO*		12 (50.0)		7 (50.0)		3 (42.9)	2 (40.0)	
Endocrine outcome								
*No*		11 (44.0)		4 (28.6)		3 (50.0)	4 (80.0)	0.159
*Yes*		14 (56.0)		10 (71.4)		3 (50.0)	1 (20.0)	
Hypothalamic dysfunction								
*No*		18 (72.0)		8 (57.1)		6 (100)	4 (80.0)	0.138
*Yes*		7 (28.0)		6 (42.9)		0	1 (20.0)	
Cognitive								
*Normal*		16 (64.0)		8 (57.1)		4 (66.7)	4 (80.0)	0.853
*Impaired*		9 (36.0)		6 (42.9)		2 (33.3)	1 (20.0)	

*n=3 IAT, n=1 atypical glioma, n=1 AG. PA, Pilocytic Astrocytoma; PMA, Pilomyxoid Astrocytoma; AG, Angiocentric Glioma; IAT, Intermediate with Anaplastic Transformation; SR, subtotal resection; D, debulking; NTR, Near total resection.

Treatment related morbidities among the cohort included visual impairment leading to visual field defect or blindness in 50% (n=12), endocrine deficits in 56% (n=14), such as cortisol deficiency, hypothyroidism, growth hormone deficiency, diabetes insipidus, or panhypopituitarism as well as cognitive impairment in 36% (n=9), such as memory loss or learning difficulties (**Table 2**). The median progression free survival (PFS) for the whole cohort was 156 months and for PMA it was 111 months that was not statistically significantly different between the groups (log-rank test, P=0.726).

### Genomewide copy number analysis of PA and PMAs

We next employed OncoScan^®^ CNV assays in order to identify genome-wide chromosomal aberrations in our 27 tumor samples. The data was analyzed using Affymetrix’s Chromosome Analysis Suite (ChAS) using the default settings. The OncoScan^®^ assays use molecular inversion probes (MIP) for the identification of genomic copy number alterations, loss of heterozygosity (LOH), and somatic mutations. It targets genome-wide chromosomal aberrations with one of the highest resolutions provided by microarrays. The interrogating MIP probes are 40 base pairs and extensively wet-validated using archived FFPE samples of major solid tumor types. It also utilizes MIPs for hotspot mutations in nine genes implicated in cancer (*BRAF, KRAS, EGFR, IDH1, IDH2, PTEN, PIK3CA, NRAS* and *TP53*). In our cohort, none of the targeted mutations on the arrays was positively detected. We also performed Sanger sequencing (SS) using standard PCR primers targeting the same mutations (**Supplementary Table 1**), that also confirmed the OncoScan results.

**Figure 2:**
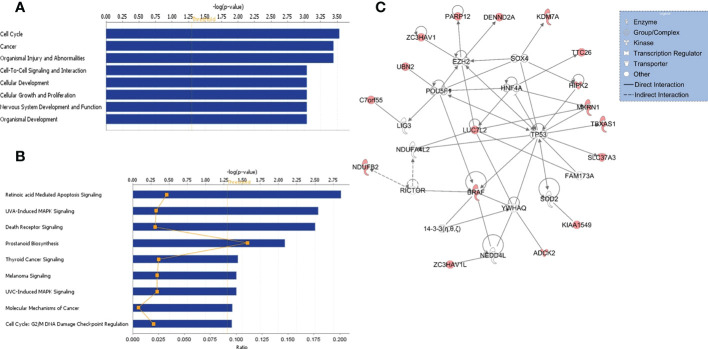
Functional/pathway and network analyses of genes in KIAA1549:BRAF fusion. Enriched biological functions **(A)** and canonical pathways **(B)** that are associated with the genes in the fusion region. The significance of function and pathway are shown in X-axis. The P value of 0.05 is indicated as the threshold line. **(C)** Gene interaction network analysis. Nodes represent genes, with their shape indicating the functional class of the gene product (see legend). The edges indicate biological relationship between the genes, with straight and dashed lines representing direct or indirect gene to gene interactions, respectively. The red color indicate genes in the fusion.

Genome-wide copy number aberration (CNA) analyses revealed 41 CNAs (34 gains (CNGs) and 7 losses (CNLs)) in all tested patients (n=17) (**Figures 1B, C**; **Table 3**). All nine PMAs harbored 20 CNAs (17 gains and 3 losses) and five PAs have 11 CNAs (all gains). Our study yielded the previously reported gain on chromosome 7q34 in over 88% of our patient cohort (89% and 80% in PMA and PA, respectively). The duplication has been previously reported in PA and known as “*KIAA1549:BRAF* fusion gene” (15, 16). Nearly all samples have similar proximal and distal end breakpoints starting from *KIAA1549* gene and reaching to *BRAF* (**Figure 1B**). The duplication outspreads nearly 1.87Mb region and comprises 9 pseudogenes, 9 uncharacterized genes, and 10 genes with OMIM ID including *KIAA1549* and *BRAF* at the breakpoints. Other genes that may also have importance in disease pathogenesis include *MKRN1, LUC7L2, UBN2, DENND2A, SLC37A3, PARP12, TBXAS1, HIPK2*, and *C7orf55* (**Figure 2**).

**Table 3:** Genomic copy number alterations (CNAs) in PA and PMA. CNAs identified in PA/PMA (archived as FFPE) samples using OncoScan arrays.

Tumor Type	Sample ID	CN	TYPE	CHRS	CYTOBAND	START	END	SIZE (bp)	#MARKER	#GENES
PMA	BT-001*	4	Gain	7	q34	138549074	140491678	1942604	222	24
		3	Gain	7	p22.3-136.3	41,420	159,118,443	159077023	11975	948
		1.5	Loss	X	p22.33-q28	177,941	155,219,364	155041423	10838	874
PMA	BT-004*	3	Gain	7	q34	138,549,074	140,491,678	1942604	222	24
		3	Gain	1	q41	221,043,002	221,724,913	681911	59	2
PMA	BT-012	1.5	Loss	8	q24.3	144,994,887	145,050,051	55164	23	2
		3	Gain	10	q11.22	47,033,943	47,702,691	668748	37	12
PMA	BT-013*	2.5	Gain	7	q34	138,549,074	140,871,571	2322497	298	25
		3	Gain	19	p13.3p11	247,231	24,544,320	24297089	2120	655
		1	Loss	1	p36.33	754,191	145,382,341	144628150	9789	1241
PMA	BT-014*	3	Gain	7	q34	138,550,993	140,499,107	1948114	224	24
PMA	BT-015*	3	Gain	7	q34	138,615,645	140,485,335	186969	207	24
PMA	BT-018*	3	Gain	7	q34	138,550,993	140,854,420	2303427	295	24
PMA	BT-019*	2.33	Gain	3	p12.3q29	75,331,108	197,852,564	122521456	8958	710
		2.44	Gain	4	p16.3p13	1,248,918	41,827,875	40578957	2629	221
		2.4	Gain	5	p15.33q35.3	38,138	180,698,312	180660174	12360	1037
		2.41	Gain	6	p25.3q27	204,908	170,913,051	170708143	12578	1196
		2.44	Gain	7	p22.3q36.3	41,420	159,118,443	159077023	11887	1142
		2.44	Gain	15	q11.12q13.3	20,161	33,073	12912	626	173
PMA	BT-034*	3	Gain	7	q34	138541150	140490207	1949057	222	24
PA	BT-002	2.67	Gain	12	p13.33q24.33	189,399	133,818,115	133628716	10358	1190
PA	BT-006*	3	Gain	7	q34	138,645,000	140,276,997	1631997	170	16
		3	Gain	22	q11.23	24,346,427	24,390,318	43891	20	4
PA	BT-020*	3	Gain	7	q34	138,561,416	140,485,335	1923919	214	23
PA	BT-021*	2.5	Gain	5	p13.1	38,138	180,698	142560	11928	989
		2.5	Gain	7	p22.3q36.3	41,420	159,118,443	159077023	11588	1108
		2.5	Gain	9	p24.3q34.3	204	141,055	140851	8472	929
		2.5	Gain	10	p15.3q26.3	463	135,434	134971	9220	892
		2.5	Gain	21	p11.2q22.3	9,648	47,707	38059	2249	289
PA	BT-028*	3.33	Gain	7	q21.3q36.3	95,430,276	159,118,443	63688167	5107	554
		3.17	Gain	11	p15.5p11.12	192,763	50,766,362	50573599	3637	510
IAT	BT-005*	4	Gain	7	q34	138,549,074	140,487,260	1938186	220	24
		1	Loss	19	p13.3	1,232,376	1,283,188	50812	29	6
IAT	BT-011*	3	Gain	7	q34	138,609,656	140,499,107	1889451	215	24
		3	Gain	8	p11.21	42,898,470	43,767,534	869064	58	4
AG	BT-010*	3.67	Gain	7	q34	138,561,416	140,499,107	1937691	221	24
		2.33	Gain	3	p13q29	70,319,707	197,852,564	127532857	9151	714
		2.84	Gain	7	q22.1q36.3	103,292,486	159,118,443	55825957	9008	403
		1.33	Loss	2	p25.3p24.2	21,493	16,743,453	16721960	1261	75
		1.33	Loss	16	p12.1p11.1	26,415,932	35,214,165	8798233	487	157
		1.33	Loss	17	p13.1p11.2	9,561,542	18,678,548	9117006	706	107

PA, Pilocytic Astrocytoma; PMA, Pilomyxoid Astrocytoma; AG, Angiocentric Glioma; IAT, Intermediate with anaplastic transformation; CN, copy number; CNA, copy number aberration; CHR, chromosome. Asterisk (*) indicates CNG in the KIAA1549:BRAF Fusion gene.

Besides the fusion gene, twelve patients have additional genomic CNAs (**Table 3**). Briefly, five patients have either partial (n=2) or full (n=3) trisomy of chromosome 7 (**Figure S1**). Interestingly, only two (BT-001 and BT-010) harbored tetrasomy of the fusion gene region (**Figure S2**). The partial trisomies in BT-10 and BT-28 are large and cover majority of the q arm. BT-013 (PMA) has a deletion on chromosome 1 (1p36.33q21.1) (**Figure S3**) and a duplication on chromosome 19 (19p13.3p11) (**Figure S4**). BT-021 has various large gains of chromosomes 5, 7, 9, 10, and 21 (**Figure S5**).

### Functional, pathway, and gene network analyses

Functional, pathway, and gene network analyses of genes within the KIAA1549:BRAF fusion region were performed using several bioinformatics tools, including DAVID (13) and IPA (QIAGEN Inc.) that indicated enrichment in cancer, cell cycle, cellular development, and nervous system development and function, and significant alterations in retinoic acid mediated apoptosis signaling, MAPK and death receptor signaling pathways (all p values < 0.01) (**Figures 2A, B**). Furthermore, gene interaction network analysis revealed hub genes that may potentially be involved in PA/PMA, including *BRAF, LUC7L2, TP53, MKRN1, RICTOR, HIPK2, HNF4A, POU5F*, and *SOX4* (**Figure 2C**).

## Discussion

Though PMA shares similar molecular features with PA, the commonest central nervous system (CNS) tumor, however, it presents more aggressive course and higher grade. Histologically, in contrast to PA, PMA does not exhibit calcification, Rosenthal fibers, and eosinophilic granule bodies (4). It mostly appears with angiocentric pattern with small and compact monomorphous piloid cells in myxoid matrix-like background. Although PMA is well characterized histologically, and similarities and differences with PA are recognized at the histological level, the studies on PMAs and differentiating both tumor types genetically are scarce. In this study, we gathered one of the largest cohorts of PA and PMA in Saudi population and investigated genomic copy number changes and outcome of these pediatric tumors.

In one of the earliest studies, Forshew et al. identified the fusion in 23 out of 26 PA samples as well as in singleton PMA (17). Gains on chromosome 5 and 6 were also detected in a few samples. Interestingly, both fusions lack the auto-inhibitory domains of BRAF and RAF1 that were interchanged by in-frame start units of the KIAA1549 and SRGAP3. This replacement creates a constitutive kinase activity. We provided a summary of the previous studies of PAs/PMAs and the identified CNAs using different array platforms (**Supplementary Table 2**).

We used Affymetrix’s OncoScan arrays that have MIPs targeting selected hot spot mutations in cancers as well as global copy number changes. Interestingly, these arrays did not identify any hot spot mutations in our patient cohort. However, we were able to identify 41 CNAs, including 34 CNGs and 7 CNLs in 17 patients tested (**Table 3**). Nine PMA samples harbor 20 CNAs (17 CNGs and 3 CNLs) and five PAs have 11 CNGs. Over 88% our patient cohort (89% in PMA and 80% in PA) have the well-defined BRAF fusion. Previous pioneering array studies, mostly done on PA tumors, indicated involvement of KIAA1549-BRAF fusion gene in low-grade brain tumors (15, 16). Alternative approaches such as RNAseq and utilization of droplet-PCR also indicated similar findings in PA (18). Such fusion is predicted to occur owing to tandem duplication events happening especially during homologous recombination of Alu elements (19–21). A large series of pilocytic astrocytoma tumors were screened for likely copy number changes using a custom designed arrays constructed with clones obtained from the Wellcome Trust Sanger Institute. The analysis indicated that 29 samples had duplication between clones RP11-355D18 and RP11-543P6 on chromosome 7. Further analysis confirmed the presence of a newly formed fusion gene between intron 16 of *KIAA1549* and intron 8 of *BRAF*. The fusion gene derives few different transcripts but essentially the fusions retain BRAF’s kinase domain (15). Under normal circumstances, BRAF’s kinase activity is regulated by NH2-terminal region that includes Ras-binding domain and is truncated in the new fusion. Once such truncation removes NH2-terminal of the *BRAF*, then regulation of fusion gene is not under autoregulation of wild type NH2-terminal but rather it is controlled by newly generated mutant NH2-terminal. Besides the fusion gene, twelve patients in our study had additional genomic CNAs (**Table 3**).

As over 88% of the samples (15 out of 17) had the gain in KIAA1549-BRAF fusion gene in our study, we investigated the gene interaction network analysis of genes in this region. The analysis indicated alterations in retinoic acid mediated apoptosis signaling, MAPK and death receptor signaling pathways and hub genes, including *BRAF, LUC7L2, MKRN1, TP53, HNF4A, RICTOR, POU5F*, *HIPK2*, and *SOX4* that may have potentially important role in tumorigenesis and cancer progression (2, 16, 22–25).

This study has potential limitations. We had a relatively small sample size of patients in each comparison group (PA, PMA, and other) and the analysis is based on a retrospective design; hence may have inherent bias. Moreover, though the KFSHRC is a referral hospital and our cohort included local as well as referred patients within Saudi Arabia, the results obtained in this study may not be generalizable to the whole population. Despite these limitations, this study is the first comprehensive study of pediatric patients with PMA and PA in Saudi population providing genome-wide copy number screening, retrospective analysis of clinicopathological features and long-term clinical outcome of these pediatric tumors. In conclusion, our study reports the first comprehensive molecular and clinicopathological analyses of one of the largest cohorts of PMA/PA samples to date in a previously unstudied population to understand the clinical features and genomic makeup of these solid types of tumors and hence may help better diagnosis and characterization of these tumors.

## Data availability statement

The dataset generated and analyzed during the current study is available at this link (https://www.dropbox.com/s/fg2vo8ebiwbc8th/Affy-Oncoscan-All-PMA-Data.rar?dl=0).

## Ethics statement

This study involved in using archived FFPE samples and was performed under IRB-approved protocols at King Faisal Specialist Hospital and Research Center (KFSHRC’s Research Advisory Council Committees including Basic Research Committee and Research Ethics Committee, RAC# 2111055). All patients were de-identified to the research team and hence the study was exempted from collecting written informed consent for their participation accordance with the institutional requirements.

## Author contributions

NK conceived and designed the experiments. NK, EAS, and DC drafted the manuscript. NK, DC, MA, and HK performed the data analyses. AA and MA involved in reviewing and editing manuscript. HA-H and AD review the pathology reports and performed pathology related tests. EAS, HA-H, AD, ANA, AA, EAM, and ZA reviewed the charts, collected clinical data, and prepared the tables related to clinical information. MA and RA performed the experiments. All authors contributed to the article and approved the submitted version.
