# Supplementary material for: A molecular study of pediatric pilomyxoid and pilocytic astrocytomas: Genome-wide copy number screening, retrospective analysis of clinicopathological features and long-term clinical outcome
Source: Front Oncol. 2023 Feb 13;13:1034292. doi: 10.3389/fonc.2023.1034292 (PMC9968872; doi:10.3389/fonc.2023.1034292)

**Supplementary Figure S1.** The figure presents all trisomy (partial or full) of chromosome 7 in five samples (BT-001, BT010, BT019, BT021, and BT028).

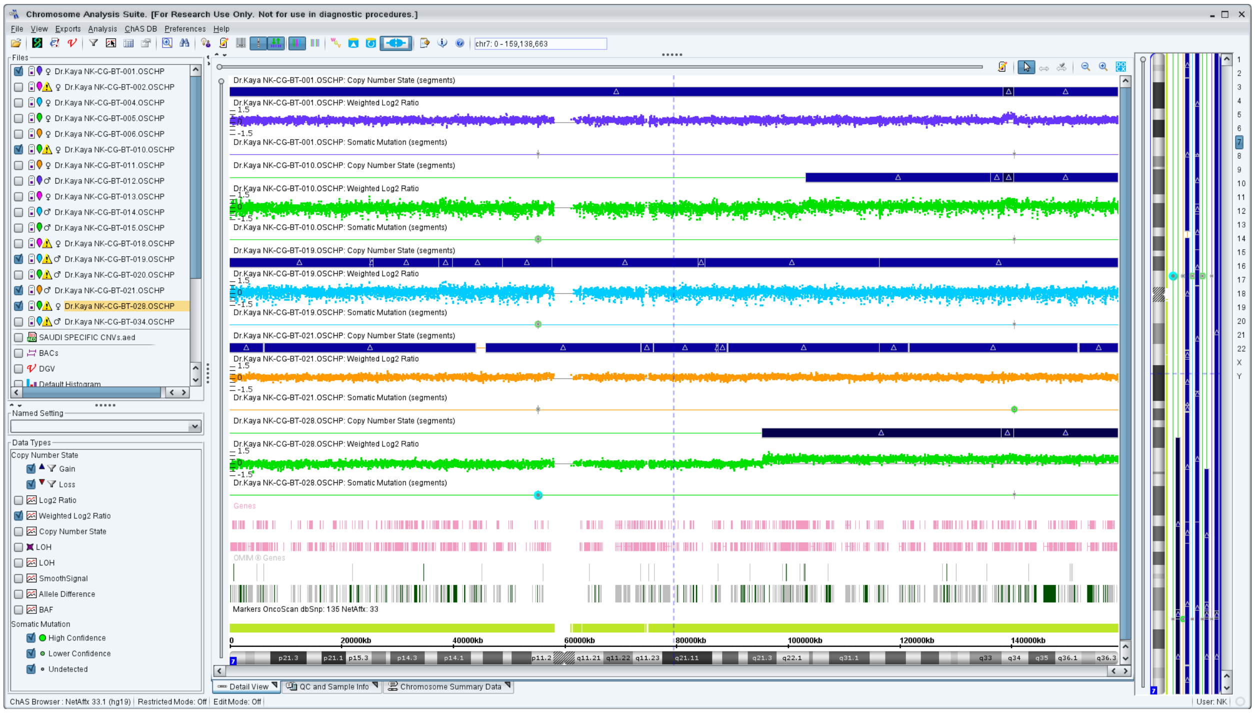

**Supplementary Figure S2.** Among these trisomy 7 samples, two samples (BT-001 and BT-010) harbored tetrasomy of the fusion gene region.

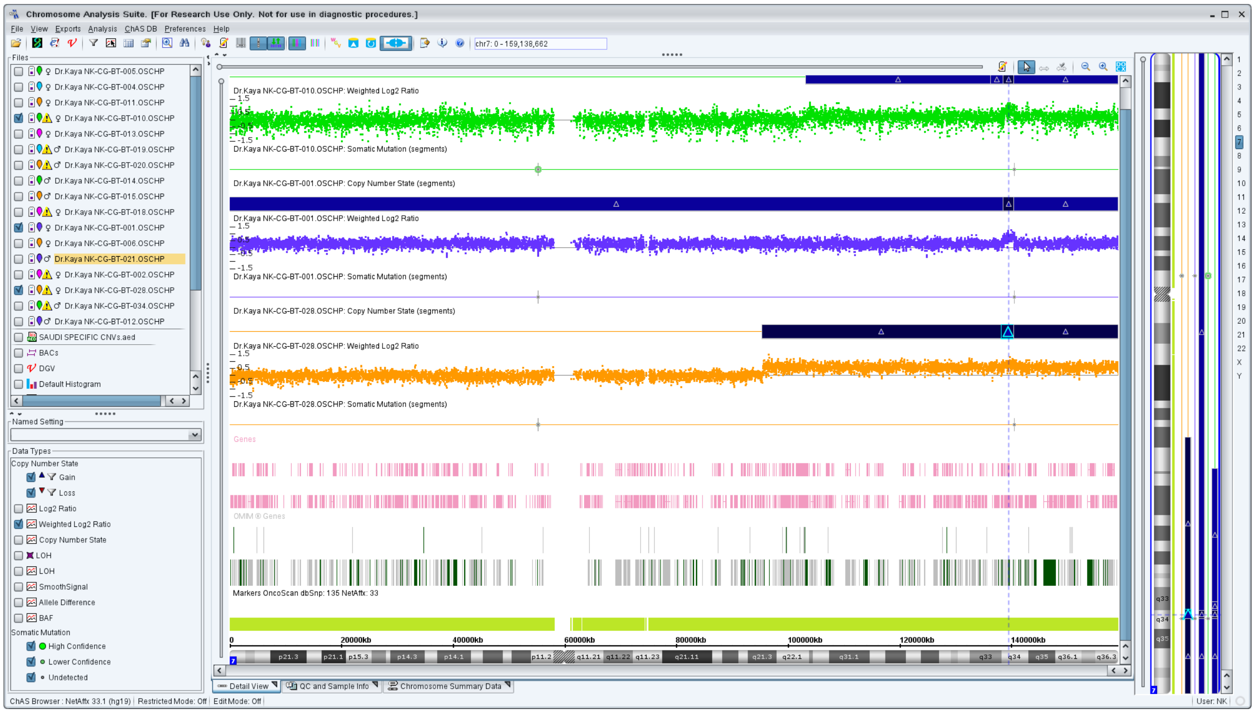

**Supplementary Figure S3.** BT-013 has a deletion extending on chromosome 1 starting from p arm reaching to q arm (1p36.33q21.1).

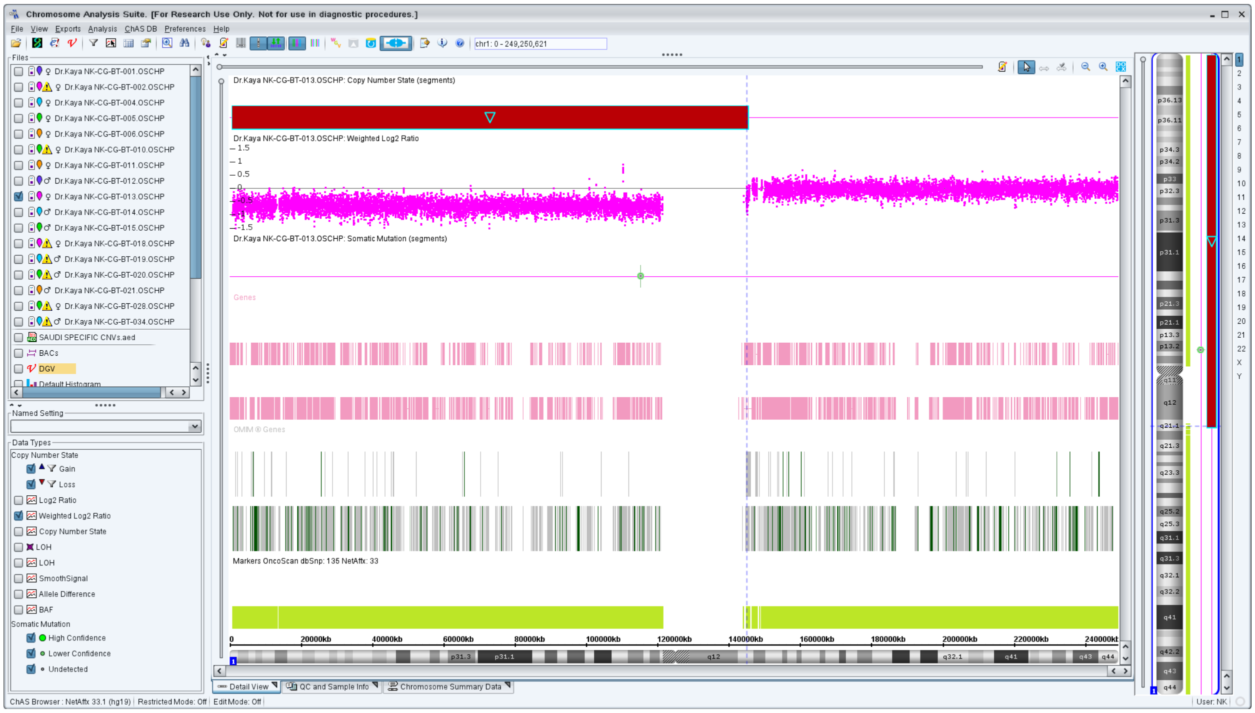

**Supplementary Figure S4.** The figure depicts duplication on chromosome 19 covering nearly whole arm and stretching between p13.3 and p11 cytobands in patient BT-013 (19p13.3p11).

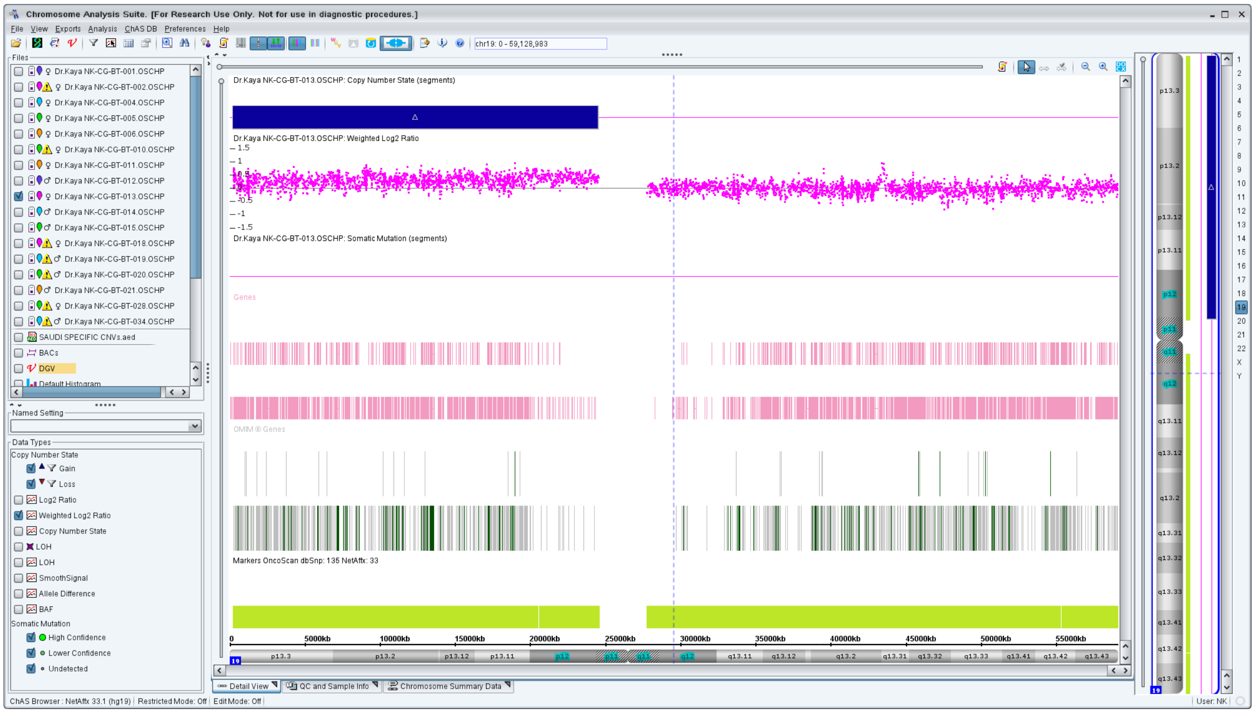

**Supplementary Figure S5.** The figure shows trisomies for different chromosomes on sample BT-021

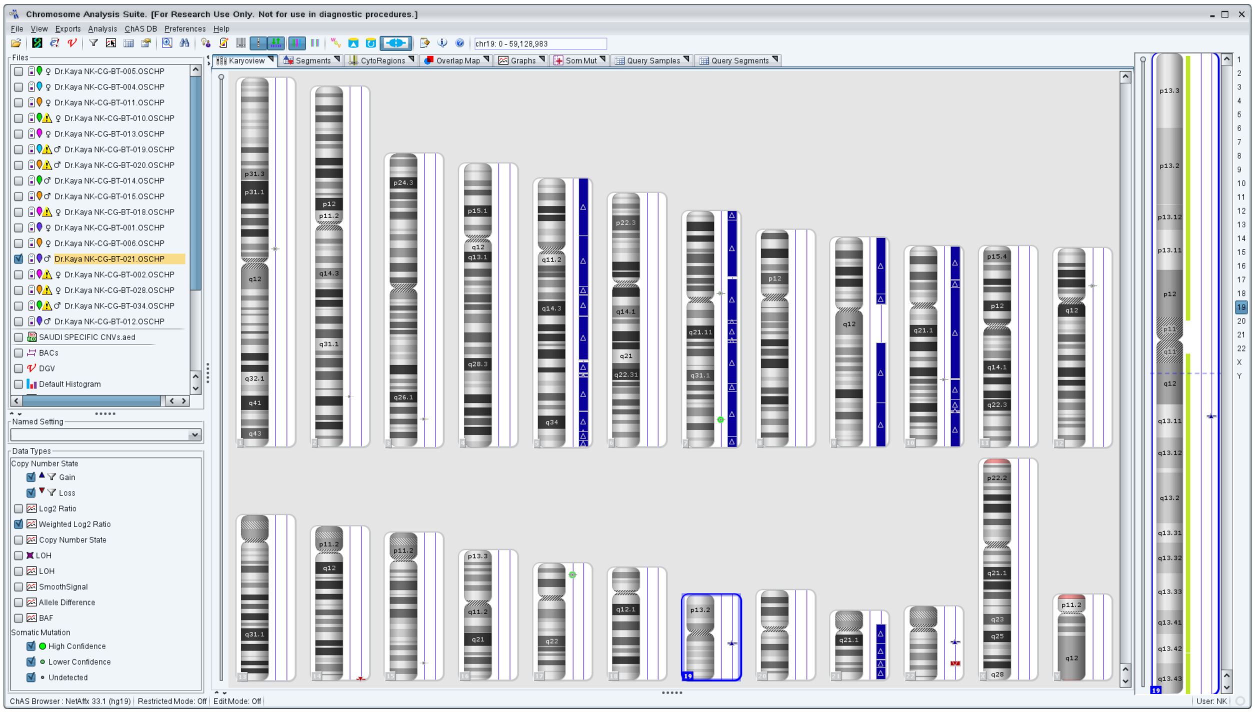

Supplement: Supplementary file 1 [file DataSheet_1.pdf]
