# Supplementary material for: A molecular study of pediatric pilomyxoid and pilocytic astrocytomas: Genome-wide copy number screening, retrospective analysis of clinicopathological features and long-term clinical outcome
Source: Front Oncol. 2023 Feb 13;13:1034292. doi: 10.3389/fonc.2023.1034292 (PMC9968872; doi:10.3389/fonc.2023.1034292)
Supplement: Supplementary file 2 [file Table_1.docx]

Supplementary Table 1. The common mutations targeted by Sanger sequencing using standard PCR.

| Change | ChromOSOME | Start | End | COSMIC ID |
| --- | --- | --- | --- | --- |
| BRAF:p.G469A:c.1406G>C | 7 | 140481401 | 140481402 | COSM460 |
| BRAF:p.G469E:c.1406G>A | 7 | 140481401 | 140481402 | COSM461 |
| BRAF:p.V600E:c.1799T>A | 7 | 140453135 | 140453136 | COSM476 |
| BRAF:p.V600K:c.1798_1799GT>AA | 7 | 140453135 | 140453137 | COSM473 |
| EGFR:p.D770_N771insSVD:c.2311_2312ins9 | 7 | 55249012 | 55249014 | COSM13428 |
| EGFR:p.E746_A750del:c.2235_2249del15 | 7 | 55242464 | 55242479 | COSM6223 |
| EGFR:p.E746_A750del:c.2236_2250del15 | 7 | 55242465 | 55242480 | COSM6225 |
| EGFR:p.E746_T751>A:c.2237_2251del15 | 7 | 55242466 | 55242481 | COSM12678 |
| EGFR:p.G719A:c.2156G>C | 7 | 55241707 | 55241708 | COSM6239 |
| EGFR:p.G719C:c.2155G>T | 7 | 55241706 | 55241707 | COSM6253 |
| EGFR:p.G719S:c.2155G>A | 7 | 55241706 | 55241707 | COSM6252 |
| EGFR:p.H773_V774insNPH:c.2319_2320ins9 | 7 | 55249020 | 55249022 | COSM12381 |
| EGFR:p.L747_E749P/del:c.2239_2248>C/G | 7 | 55242468 | 55242478 | COSM12382\|\|COSM6218 |
| EGFR:p.L747_P753>S:c.2240_2257del18 | 7 | 55242469 | 55242487 | COSM12370 |
| EGFR:p.L747_T751del:c.2240_2254del15 | 7 | 55242469 | 55242484 | COSM12369 |
| EGFR:p.L858R:c.2573T>G | 7 | 55259514 | 55259515 | COSM6224 |
| EGFR:p.L861Q:c.2582T>A | 7 | 55259523 | 55259524 | COSM6213 |
| EGFR:p.T790M:c.2369C>T | 7 | 55249070 | 55249071 | COSM6240 |
| EGFR:p.V769_D770insASV:c.2307_2308ins9 | 7 | 55249008 | 55249010 | COSM12376 |
| IDH1:p.R132H:c.395G>A | 2 | 209113111 | 209113112 | COSM28746 |
| IDH2:p.R140Q:c.419G>A | 15 | 90631933 | 90631934 | COSM41590 |
| IDH2:p.R172K:c.515G>A | 15 | 90631837 | 90631838 | COSM33733 |
| KRAS:p.A146P:c.436G>C | 12 | 25378561 | 25378562 | COSM19905 |
| KRAS:p.G12A:c.35G>C | 12 | 25398283 | 25398284 | COSM522 |
| KRAS:p.G12C/S:c.34G>T/A | 12 | 25398284 | 25398285 | COSM516\|\|COSM517 |
| KRAS:p.G12D/V:c.35G>A/T | 12 | 25398283 | 25398284 | COSM521\|\|COSM520 |
| KRAS:p.G13D:c.38G>A | 12 | 25398280 | 25398281 | COSM532 |
| KRAS:p.Q61H:c.183A>C | 12 | 25380274 | 25380275 | COSM554 |
| KRAS:p.Q61H:c.183A>T | 12 | 25380274 | 25380275 | COSM555 |
| KRAS:p.Q61K/K:c.180_181TC>TA/AA | 12 | 25380276 | 25380278 | COSM549\|\|COSM87298 |
| NRAS:p.G12D:c.35G>A | 1 | 115258746 | 115258747 | COSM564 |
| NRAS:p.G12S/C:c.34G>A/T | 1 | 115258747 | 115258748 | COSM563\|\|COSM562 |
| NRAS:p.G12V:c.35G>T | 1 | 115258746 | 115258747 | COSM566 |
| NRAS:p.Q61K:c.181C>A | 1 | 115256529 | 115256530 | COSM580 |
| NRAS:p.Q61L:c.182A>T | 1 | 115256528 | 115256529 | COSM583 |
| NRAS:p.Q61R:c.182A>G | 1 | 115256528 | 115256529 | COSM584 |
| PIK3CA:p.E542K:c.1624G>A | 3 | 178936081 | 178936082 | COSM760 |
| PIK3CA:p.E545K:c.1633G>A | 3 | 178936090 | 178936091 | COSM763 |
| PIK3CA:p.H1047L:c.3140A>T | 3 | 178952084 | 178952085 | COSM776 |
| PIK3CA:p.H1047R:c.3140A>G | 3 | 178952084 | 178952085 | COSM775 |
| PIK3CA:p.Q546K:c.1636C>A | 3 | 178936093 | 178936094 | COSM766 |
| PTEN:p.K267fs*9:c.800delA | 10 | 89717774 | 89717775 | COSM5809 |
| PTEN:p.P248fs*5:c.741_742insA | 10 | 89717715 | 89717717 | COSM4986 |
| PTEN:p.R130*:c.388C>T | 10 | 89692903 | 89692904 | COSM5152 |
| PTEN:p.R130G:c.388C>G | 10 | 89692903 | 89692904 | COSM5219 |
| PTEN:p.R130Q/fs*4:c.389G>A/delG | 10 | 89692904 | 89692905 | COSM5033\|\|COSM5817 |
| PTEN:p.R159S:c.477G>T | 10 | 89692992 | 89692993 | COSM5287 |
| PTEN:p.R233*:c.697C>T | 10 | 89717671 | 89717672 | COSM5154 |
| TP53:p.C176F:c.527G>T | 17 | 7578402 | 7578403 | COSM10645 |
| TP53:p.G245S/C:c.733G>A/T | 17 | 7577547 | 7577548 | COSM6932\|\|COSM11081 |
| TP53:p.H179R:c.536A>G | 17 | 7578393 | 7578394 | COSM10889 |
| TP53:p.R175H:c.524G>A | 17 | 7578405 | 7578406 | COSM10648 |
| TP53:p.R196*:c.586C>T | 17 | 7578262 | 7578263 | COSM10705 |
| TP53:p.R213*:c.637C>T | 17 | 7578211 | 7578212 | COSM10654 |
| TP53:p.R248Q/L:c.743G>A/T | 17 | 7577537 | 7577538 | COSM10662\|\|COSM6549 |
| TP53:p.R248W:c.742C>T | 17 | 7577538 | 7577539 | COSM10656 |
| TP53:p.R249S:c.747G>T | 17 | 7577533 | 7577534 | COSM10817 |
| TP53:p.R273C/S:c.817C>T/A | 17 | 7577120 | 7577121 | COSM10659\|\|COSM43909 |
| TP53:p.R273H/L:c.818G>A/T | 17 | 7577119 | 7577120 | COSM10660\|\|COSM10779 |
| TP53:p.R282W:c.844C>T | 17 | 7577093 | 7577094 | COSM10704 |
| TP53:p.R306*:c.916C>T | 17 | 7577021 | 7577022 | COSM10663 |
| TP53:p.V157F:c.469G>T | 17 | 7578460 | 7578461 | COSM10670 |
| TP53:p.Y163C:c.488A>G | 17 | 7578441 | 7578442 | COSM10808 |
| TP53:p.Y220C:c.659A>G | 17 | 7578189 | 7578190 | COSM10758 |
