# Supplementary material for: A molecular study of pediatric pilomyxoid and pilocytic astrocytomas: Genome-wide copy number screening, retrospective analysis of clinicopathological features and long-term clinical outcome
Source: Front Oncol. 2023 Feb 13;13:1034292. doi: 10.3389/fonc.2023.1034292 (PMC9968872; doi:10.3389/fonc.2023.1034292)
Supplement: Supplementary file 3 [file Table_2.docx]

| **#Patients** | **Tumor Type** | **Chromosome** | **CN status** | **Fusion Genes** | **Detection method** | **Refs** |
| --- | --- | --- | --- | --- | --- | --- |
| 37 | PA | 7q34 gain | Gain | KIAA1549:BRAF Fusion | FISH | [(Becker et al., 2015)](#RANGE!_ENREF_2) |
| 27 | PA | 7q34 normal | Normal |  |  |  |
| 95 | PA | 7q34 gain | Gain | KIAA1549–BRAF | aCGH/ FISH | [(Cin et al., 2011)](#RANGE!_ENREF_3) |
| 2 | PA | 7q34 deletion | Deletion | FAM131B–BRAF fusion |  |  |
| 2 | PA | 3p25.3-p25.2 | ? | SRGAP3–RAF1 fusion |  |  |
| 23 | PA | 7q34 | Gain | KIAA1549–BRAF | SNP arrays | [(Forshew et al., 2009)](#RANGE!_ENREF_4) |
| 2 | PA |  | Normal |  |  |  |
| 1 | PA | 3p25 | Gain | KIAA1549–BRAF |  |  |
| 1 | PMA | 7q34 | Gain | KIAA1549–BRAF |  |  |
| 3 | PMA | 7q34 | Gain | KIAA1549–BRAF | FISH/ Oncoscan SNP arrays | [(Ho et al., 2015)](#RANGE!_ENREF_5) |
| 1 | PMA | 7q34 | Normal | KIAA1549–BRAF |  |  |
| 7 | PA | 7q34 | Gain | KIAA1549–BRAF |  |  |
| 1 | PA | Chr7 | Trisomy | NI |  |  |
| 1 | PA | 9p21.3 | deletion | NI |  |  |
| 3 | PA | 7q34/ 9p21.3 | Normal | NI |  |  |
| 17 | PA | 7q34 | Gain | KIAA1549:BRAF | SNP arrays | [(Sievert et al., 2009)](#RANGE!_ENREF_10) |
| 5 | PA | 7q34 | Normal | KIAA1549:BRAF |  |  |
| 7 | PA | 7q34 | Normal | NI | aCGH | [(Bar et al., 2008)](#RANGE!_ENREF_1) |
| 12 | PA | 7q34 | Gain | NI |  |  |
| 1 | PA | Chr7/ 7q34 | Gain / Normal | NI |  |  |
| 1 | PA | Chr5,6 and 7/ 7q34 | Gain/ Gain | NI |  |  |
| 2 | PA | Chr17, 19 and 22 / 7q34 | Loss/Gain | NI |  |  |
| 1 | PA | Chr19/ 7q34 | Loss/Gain | NI |  |  |
| 1 | PA | Chr5 and 6/ 7q34 | Gain/ Gain | NI |  |  |
| 1 | PA | Chr16, 17, 19 and 22 / 7q34 | Loss/ Gain | NI |  |  |
| 3 | PMA | 8q24 | Gain | NI | aCGH | [(Jeon et al., 2008)](#RANGE!_ENREF_7) |
| 3 | PMA | 8p23.3 | Loss | NI |  |  |
| 4 | PMA | 9p24.3 | Loss | NI |  |  |
| 4 | PMA | 15q26.3 | Loss | NI |  |  |
| 12 | PA | 8q24 | Gain | NI |  |  |
| 4 | PA | 10p15.3 | Gain | NI |  |  |
| 4 | PA | 8p23.3 | Loss | NI |  |  |
| 5 | PA | 9p24.3 | Loss | NI |  |  |
| 5 | PA | 15q26.3 | Loss | NI |  |  |
| 1 | PA | 17q33q34 | Loss | KIAA1549:BRAF Fusion | SNP arrays | [(Roth et al., 2015)](#RANGE!_ENREF_9) |

Supplementary Table 2. Previously identified copy number alterations found in pilocytic and pilomyxoid astrocytomas using different array platforms.

REFERENCES

BAR, E. E., LIN, A., TIHAN, T., BURGER, P. C. & EBERHART, C. G. 2008. Frequent gains at chromosome 7q34 involving BRAF in pilocytic astrocytoma. *J Neuropathol Exp Neurol,* 67**,** 878-87.

BECKER, A. P., SCAPULATEMPO-NETO, C., CARLONI, A. C., PAULINO, A., SHEREN, J., AISNER, D. L., MUSSELWHITE, E., CLARA, C., MACHADO, H. R., OLIVEIRA, R. S., NEDER, L., VARELLA-GARCIA, M. & REIS, R. M. 2015. KIAA1549: BRAF Gene Fusion and FGFR1 Hotspot Mutations Are Prognostic Factors in Pilocytic Astrocytomas. *J Neuropathol Exp Neurol,* 74**,** 743-54.

CIN, H., MEYER, C., HERR, R., JANZARIK, W. G., LAMBERT, S., JONES, D. T., JACOB, K., BENNER, A., WITT, H., REMKE, M., BENDER, S., FALKENSTEIN, F., VAN ANH, T. N., OLBRICH, H., VON DEIMLING, A., PEKRUN, A., KULOZIK, A. E., GNEKOW, A., SCHEURLEN, W., WITT, O., OMRAN, H., JABADO, N., COLLINS, V. P., BRUMMER, T., MARSCHALEK, R., LICHTER, P., KORSHUNOV, A. & PFISTER, S. M. 2011. Oncogenic FAM131B-BRAF fusion resulting from 7q34 deletion comprises an alternative mechanism of MAPK pathway activation in pilocytic astrocytoma. *Acta Neuropathol,* 121**,** 763-74.

FORSHEW, T., TATEVOSSIAN, R. G., LAWSON, A. R., MA, J., NEALE, G., OGUNKOLADE, B. W., JONES, T. A., AARUM, J., DALTON, J., BAILEY, S., CHAPLIN, T., CARTER, R. L., GAJJAR, A., BRONISCER, A., YOUNG, B. D., ELLISON, D. W. & SHEER, D. 2009. Activation of the ERK/MAPK pathway: a signature genetic defect in posterior fossa pilocytic astrocytomas. *J Pathol,* 218**,** 172-81.

HO, C. Y., MOBLEY, B. C., GORDISH-DRESSMAN, H., VANDENBUSSCHE, C. J., MASON, G. E., BORNHORST, M., ESBENSHADE, A. J., TEHRANI, M., ORR, B. A., LAFRANCE, D. R., DEVANEY, J. M., MELTZER, B. W., HOFHERR, S. E., BURGER, P. C., PACKER, R. J. & RODRIGUEZ, F. J. 2015. A clinicopathologic study of diencephalic pediatric low-grade gliomas with BRAF V600 mutation. *Acta Neuropathol,* 130**,** 575-85.

JACOB, K., ALBRECHT, S., SOLLIER, C., FAURY, D., SADER, E., MONTPETIT, A., SERRE, D., HAUSER, P., GARAMI, M., BOGNAR, L., HANZELY, Z., MONTES, J. L., ATKINSON, J., FARMER, J. P., BOUFFET, E., HAWKINS, C., TABORI, U. & JABADO, N. 2009. Duplication of 7q34 is specific to juvenile pilocytic astrocytomas and a hallmark of cerebellar and optic pathway tumours. *Br J Cancer,* 101**,** 722-33.

JEON, Y. K., CHEON, J. E., KIM, S. K., WANG, K. C., CHO, B. K. & PARK, S. H. 2008. Clinicopathological features and global genomic copy number alterations of pilomyxoid astrocytoma in the hypothalamus/optic pathway: comparative analysis with pilocytic astrocytoma using array-based comparative genomic hybridization. *Mod Pathol,* 21**,** 1345-56.

LIN, A., RODRIGUEZ, F. J., KARAJANNIS, M. A., WILLIAMS, S. C., LEGAULT, G., ZAGZAG, D., BURGER, P. C., ALLEN, J. C., EBERHART, C. G. & BAR, E. E. 2012. BRAF alterations in primary glial and glioneuronal neoplasms of the central nervous system with identification of 2 novel KIAA1549:BRAF fusion variants. *J Neuropathol Exp Neurol,* 71**,** 66-72.

ROTH, J. J., SANTI, M., POLLOCK, A. N., HARDING, B. N., RORKE-ADAMS, L. B., TOOKE, L. S. & BIEGEL, J. A. 2015. Chromosome band 7q34 deletions resulting in KIAA1549-BRAF and FAM131B-BRAF fusions in pediatric low-grade Gliomas. *Brain Pathol,* 25**,** 182-92.

SIEVERT, A. J., JACKSON, E. M., GAI, X., HAKONARSON, H., JUDKINS, A. R., RESNICK, A. C., SUTTON, L. N., STORM, P. B., SHAIKH, T. H. & BIEGEL, J. A. 2009. Duplication of 7q34 in pediatric low-grade astrocytomas detected by high-density single-nucleotide polymorphism-based genotype arrays results in a novel BRAF fusion gene. *Brain Pathol,* 19**,** 449-58.
